# **α**V**β**5 and CD44 Are Oxygen-Regulated Human Embryonic Stem Cell Attachment Factors

**DOI:** 10.1155/2013/729281

**Published:** 2013-12-26

**Authors:** Deepak Kumar, Saniya Gupta, Ying Yang, Nicholas R. Forsyth

**Affiliations:** Guy Hilton Research Centre, Institute of Science and Technology in Medicine, University of Keele, Thornburrow Drive, Hartshill, Stoke-on-Trent, Staffordshire ST4 7QB, UK

## Abstract

Human embryonic stem cells (hESCs) have great potential for clinical therapeutic use. However, relatively little is known of the mechanisms which dictate their specificity of adhesion to substrates through adhesion proteins including integrins. Previous observations demonstrated enhanced clonogenicity in reduced oxygen culture systems. Here, we demonstrated via antibody blocking experiments that **α**V**β**5 and **α**6 significantly promoted hESC attachment in 2% O_2_ only, whereas blockage of CD44 inhibited cell attachment in 21% O_2_ alone. Immunofluorescence confirmed expression of **α**V**β**5 and CD44 in both 2% O_2_ and 21% O_2_ cultured hESCs while flow cytometry revealed significantly higher **α**V**β**5 expression in 2% O_2_ versus 21% O_2_ cultured hESCs and higher CD44 expression in 21% O_2_ versus 2% O_2_ cultured hESCs. Adhered hESCs following blockage of **α**V**β**5 in 2% O_2_ displayed a reduction in nuclear colocalisation of Oct-4 and Nanog with little effect observed in 21% O_2_. Blockage of CD44 had the converse effect with dramatic reductions in nuclear colocalisation of Oct-4 and Nanog in 21% O_2_ cultured hESC which retained adherence, but not in 2% O_2_ cultured cells. Identification of oxygen-dependent substrate attachment mechanisms in hESCs has the potential to play a role in the development of novel substrates to improve hESC attachment and culture.

## 1. Introduction

Human embryonic stem cells (hESCs), derived from the inner cell mass of preimplantation blastocysts, have an inherent capacity for indefinite self-renewal [1]. Due to their differentiation capacity, immortality and immunological privilege, hESCs hold great promise for clinical therapeutics when used in combination with tissue engineering and regenerative medicine approaches [1–3]. The single currently active approved clinical safety trial incorporating hESCs is for age-related macular degeneration [4, 5].

Typical *in vitro* expansion of hESCs involves either direct coculture with mitotically inactivated mouse (or human) embryonic fibroblasts (MEFs) or feeder-free methods, where preconditioned media and biological substrates such as Matrigel are employed [1, 6]. Matrigel, a loosely defined gel sourced from Engelbreth-Holm-Swarm tumours, is comprised of extracellular matrix (ECM) proteins including laminin-111, collagen IV, heparin sulphate proteoglycans, entactin, fibronectin, growth factors, matrix-degrading enzymes and their inhibitors, and other yet to be defined components [6]. Limitations of culturing hESCs using Matrigel (and other biological substrates) include batch to batch variability, xenogenic contamination, expression of foreign oligosaccharide residues, and scale-up issues [7, 8]. Alternatives include collagen IV, fibronectin, laminin, vitronectin [9], recombinant vitronectin [8], human serum containing medium conditioned by human embryonic fibroblasts derived from hESCs [10], and hyaluronic acid hydrogels [11].

hESCs ECM attachment is primarily mediated by integrins (heterodimeric, transmembrane glycoproteins) and other surface receptors [12]. The integrin family, comprised of 18 alpha (*α*) subunits and 8 beta (*β*) subunits, has 24 recognised distinct heterodimer arrangements each with a specific set of functions [12–14]. Integrin functions include mediating cell-cell, cell-ECM, and cytoskeletal-ECM interactions. ECM proteins essential for hESC adhesion and pluripotency retention include laminin-111, collagen IV, fibronectin, and vitronectin [15]. Laminin is an essential component of virtually all basement membranes [16]. Laminin functions include the mediation of cell adhesion, cell spreading, cell migration, and cell proliferation. The laminin-specific integrin receptor is *α*6*β*1 while *α*1*β*1, *α*2*β*1, and *α*3*β*1 are also recognised in a non-specific manner. Integrin subunits and heterodimers detected on the surface of hESCs include *α*2, *α*3, *α*5, *α*6, *α*11, *β*1, and *α*V*β*5. These subunits can heterodimerise to form receptors for fibronectin (*α*5*β*1), vitronectin (*α*V*β*5), collagen and laminin (*α*2*β*1), laminin-111 (*α*6*β*1), and collagen, laminin, and VCAM1 (*α*9*β*1) [8, 17–19]. Antibody-directed blockage of the *α*5*β*1 heterodimer impacted hESC attachment across a range of defined substrate coatings including collagen IV, laminin, and entactin, when cultured with MEF-conditioned media, demonstrating that fibronectin was secreted by feeder cells which subsequently adsorbed onto surfaces promoting hESC adherence [8]. In defined media (mTeSR1), blocking *α*5*β*1 had no effect on hESC attachment to a vitronectin-coated substrate but hindered adhesion to all other ECM protein substrates (laminin, entactin, and collagen IV), suggesting that hESC substrate adhesion via *α*V*β*5 was essential for expansion of hESCs when cultured using defined media [8].

To this point, however, there are very few reports which have explored the role, if any, that oxygen concentration has on integrin expression level in hESC. Reports from mouse embryonic stem cells (*β*1), human mesenchymal stem cells (hMSC) (*α*1, *α*3, *α*5, *α*6, *α*11, *α*V, *β*1, and *β*3), and chondrocytes (*β*1) have indicated that specific integrin subunits, indicated in parentheses, display an oxygen sensitivity, hypoxia inducible factor 1-dependent in some instances, resulting in selected increased transcriptional expression of indicated subunits [20, 21]. Reports have also indicated that reduced oxygen culture has resulted in selected transcriptional downregulation of integrin subunits, indicated in parentheses, in hMSC (*α*2), trophoblast (*α*1, *α*4, and *α*5), breast carcinoma cells (*α*5), gastric cancer cells (*α*5), cytotrophoblasts (*α*6), and melanoma cell lines (*α*V, *β*1, and *α*V*β*3) [22–28].

Previous studies of the effects of reduced oxygen on hESC culture have demonstrated enhanced clonogenicity, reduced chromosomal aberration frequency, and improved consistency of embryoid body formation, significant transcriptional alterations, and permissive single-cell derived progenitor isolation [29, 30]. A previous study by our group described reduced transcriptional heterogeneity between hESC lines (H1, H9, and RH1) cultured in physiological normoxia which was accompanied by significant upregulation, but with modest fold change differences, of specific integrin subunits (*α*6, *α*E, *α*V, and *β*5) in comparison to hyperoxia (21% O_2_) [31]. We have now extended these observations and described the translational consequences of physiological oxygen on integrin subunit dependency and CD44 reliance for adhesion and pluripotent marker expression, in hESC (SHEF1). We demonstrate that adhesion, pluripotent marker expression and localisation in hESCs were *α*V*β*5 dependent in 2% O_2_ and CD44 dependent in 21% O_2_. These findings will help drive the future development of novel substrates to improve hESC attachment and expansion during *in vitro* expansion.

## 2. Materials and Methods

### 2.1. Human Embryonic Stem Cell Culture

Conditioned culture media were prepared using mouse embryonic fibroblasts (MEFs) as previously described [6]. In brief, hESC media comprised Knock-out DMEM (KO-DMEM) (Gibco-Invitrogen, UK) supplemented with 20% Knock-out Serum Replacement (Gibco-Invitrogen, UK), 1% L-glutamine (Lonza, UK), 1% nonessential amino acids (Lonza, UK), 4 ng/mL basic fibroblastic growth factor (Lonza, UK), and 0.1 mM *β*-mercaptoethanol (Gibco-Invitrogen, UK). hESC media were conditioned overnight on semiconfluent MEFs and then further supplemented with 4 ng/mL of bFGF and sterile filtered (Millipore, Watford, UK) before use [6]. hESCs (SHEF1) were cultured on Matrigel (BD Biosciences, Oxford, UK) coated flasks in two different oxygen tensions; 2% O_2_ (using the SCI-TIVE workstation; Ruskinn, Pencoed, UK) and 21% O_2_ (Heraeus Cytoperm 2 incubator; Thermo Electron Corporation, UK). Media were changed on a daily basis and cells passaged every 2-3 days after reaching 90% confluence using a brief 0.25% trypsin and EDTA treatment for 1-2 minutes at room temperature, followed by centrifugation for 3 minutes at 1200 rpm and replated at a 1 : 2 ratio.

### 2.2. Integrin Blocking and Cell Attachment

hESCs were pretreated with blocking antibodies raised against integrin subunits including anti-integrin *α*V (R&D Biosystems, Abingdon, UK), anti-integrin *α*V*β*5 (Chemicon International, Watford, UK), anti-integrin *β*5 (R&D Biosystems, Abingdon, UK), anti-integrin *α*E (Lifespan Bioscience, Nottingham UK), anti-integrin *α*6 (Autogen Bioclear, Calne, UK), and anti-CD44 (HCAM) (Santa Cruz Biotechnology, Heidelberg, Germany). hESCs were incubated with either 0, 1, or 25 *μ*g/mL concentrations of antibody (in PBS) in either 2% O_2_ or 21% O_2_ at 37°C for 30 minutes in KO-DMEM. Cells were then re-plated into Matrigel coated 6-well plates at a density of 4 × 10^5^ cells per well and incubated at either 2% O_2_ or 21% O_2_ for 24 hours. After 24 hours, cells were trypsinised (as described above) and counts recorded with a haemocytometer. Cell viability and nontoxicity of antibody solution were determined by staining hESCs with Trypan Blue at a 1 : 1 ratio to cell solution.

### 2.3. Immunofluorescence Staining

hESCs (400 000 cells/well) were seeded onto Matrigel coated 24-well plates and expanded to approximately 70% confluence in both oxygen concentrations (2% O_2_ and 21% O_2_). Media were removed and the cells were fixed in 4% paraformaldehyde (in PBS) for 40 minutes. Cells were washed with PBS and nonspecific proteins were blocked with 3% bovine serum albumin for 1 hour at room temperature. Cells were incubated with 50 *μ*g/mL anti-*α*V*β*5 anti-human monoclonal antibody solution at 4°C overnight. Primary antibody binding was visualised by incubation with donkey anti-human IgG (NL557; R&D Biosystems), at 5 *μ*g/mL for 2 hours at room temperature. Nuclei were visualised by counterstaining with DAPI and mounted with Vectashield (Vector Laboratories, Burlingame, CA). Images were recorded on a fluorescent microscope (Nikon TZ1; Leica, Germany).

To assess expression of pluripotency-associated proteins after antibody blocking, cells were first fixed (as above) and subsequently incubated with 1 *μ*g/100 *μ*L of either goat anti-Nanog, goat anti-Oct-3/4, mouse anti-alkaline phosphatase, or mouse anti-SSEA-4 monoclonal antibody solutions (SC008; R&D Biosystems, UK) at 4°C overnight. Nanog and Oct 3/4 binding were visualised with donkey anti-goat IgG (NL003; R&D Biosystems) and alkaline phosphatase and SSEA-4 were visualised with goat anti-mouse IgG (NL557; R&D Biosystems), 5 *μ*g/mL for 2 hours at room temperature. Nuclei were visualised by counterstaining with DAPI and mounted using Vectashield (Vector Laboratories, Burlingame, CA). Images were recorded on a fluorescent microscope (Nikon TZ1; Leica, Germany). To quantify the nuclear association of Oct-4 and Nanog expression, images were captured from five independent fields using both the DAPI and relevant fluorochrome filters. Fields of view were selected randomly and colocalised DAPI and either Oct-4 or Nanog identified and expressed as a percentage of total DAPI labelled nuclei.

### 2.4. Flow Cytometry

hESCs were trypsinised and fixed in 4% paraformaldehyde in PBS for 30 minutes and then resuspended in Fluorescence activated cell sorting (FACS) buffer (0.5% foetal bovine serum in PBS). Cells were treated with 50 *μ*g/mL *α*V*β*5 and 2 *μ*g/mL CD44 (1 : 500) antibodies for 2 hours at room temperature, followed by two brief PBS washes and incubation with goat anti-mouse IgG secondary antibody (NL557; R&D Biosystems) at 5 *μ*g/mL for a further 2 hours at room temperature. Finally, hESCs were washed in PBS and resuspended in FACS buffer before analysis on a FACS flow cytometer (Beckton Dickinson, Oxfordshire, UK). Data analysis was performed with the CellQuest Software package (BD Biosciences, UK).

### 2.5. Statistical Analysis

Error bars on graphs indicate standard deviations (SD). Student's *t*-test was performed and in this study significance levels are indicated according to the legend *P < 0.05, **P < 0.01, and ***P < 0.001.

## 3. Results

### 3.1. Integrin Subunit Gene Expression in hESC under Differing Oxygen Concentrations

Previous reports have detailed widespread transcriptional alterations as a consequence of culturing hESC in reduced oxygen environments [30, 34]. We performed a further analysis of our existing data set to determine the expression levels of integrin subunits, specifically (see Supplementary Data in Supplementary Material available online at http://dx.doi.org/10.1155/2013/729281). Data revealed that integrin subunits, *α*D (*P* < 0.05); *β*1 binding protein, *β*3 binding protein, *β*4 binding protein, *α*5, *α*9, and *β*1 (all *P* < 0.01); and *α*6, *α*E, *α*V, and *β*5 (all *P* < 0.001), were expressed significantly higher in hESCs cultured in 2% O_2_ when compared to 21% O_2_ (Supplementary Figure  1). The order of relative intensity fold change (FC) with significance of 2% O_2_ over 21% O_2_ cultured hESCs was *α*D (1.81 FC), *α*V (1.64), *α*9 (1.54 FC), *α*5 (1.35 FC), *α*6 and *α*E (1.31 FC), *β*4 binding protein (1.26 FC), *β*1 and *β*1 binding protein 1 (1.20 FC), and *β*5 (1.16 FC) and *β*3 binding protein (1.09 FC) (Supplementary Table  1). Importantly, with one exception, (*α*D) all significant expression changes were associated with FC's of <2 and would, therefore, lie out with the conventional and arbitrary remit of inclusion in microarray-based analysis [32]. In this instance we elected to perform a nonbiased inclusion approach where significant changes in expression between parameters (21% O_2_ and 2% O_2_) were the solitary inclusion criteria.

### 3.2. The Effect of Receptor Blocking on hESC Attachment

**Figure 1:**
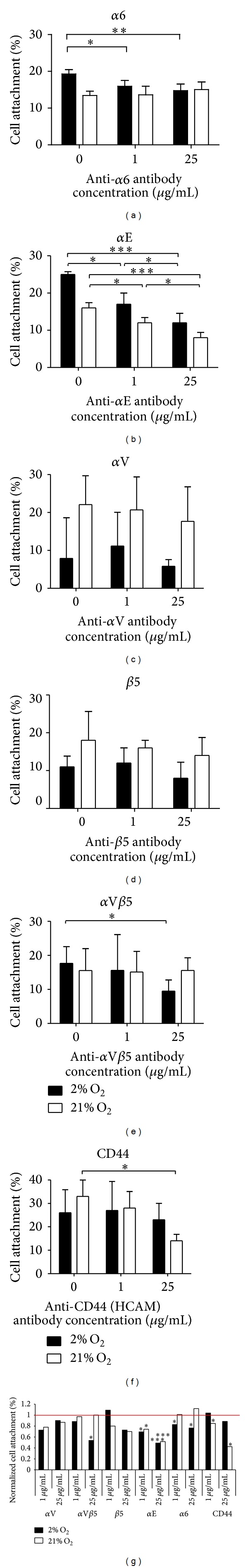
Integrin blocking effects on hESCs attachment. hESCs cultured in 2% O_2_ or 21% O_2_ and preincubated with anti- (a) *α*6, (b) *α*E, (c) *α*V, (d) *β*5, (e) *α*V*β*5, and (f) CD44 antibodies. (*n* = 6); *P < 0.05, **P < 0.01, and ***P < 0.001. *y*-axis indicates % of input cell attachment 24 hours after antibody blocking. Black and open bars indicate % of cell attachment in 2% O_2_ and 21% O_2_ after antibody blocking treatment, respectively. Error bars indicate standard deviations (SD). (g) Cell attachment data normalised to unblocked controls. Asterisks indicate significant differences to unblocked controls. Values indicate mean percentage of cell attachment (*n* = 6); *P < 0.05, **P < 0.01, and ***P < 0.001.

Integrin subunits, *α*6, *α*E, *α*V, and *β*5, displayed highly significant changes in expression between 2% O_2_ and 21% O_2_ and were selected for evaluation in a cell attachment study. CD44 (Hyaluronan receptor) was selected based on our earlier observations which described significantly reduced FACS detection of CD44 in 2% O_2_ versus 21% O_2_ cultured H1and H9 hESC [33]. Receptor blocking using specific antibodies was performed to determine the effect on hESC adhesion in both 2% O_2_ and 21% O_2_ (Figure 1). Blockage of *α*6 significantly reduced hESC attachment to Matrigel in 2% O_2_ with increasing antibody concentration but had no observed effect in 21% O_2_ (Figure 1(a)). Blocking of *α*E significantly hindered hESC attachment to Matrigel, in both oxygen environments (2% and 21% O_2_) (Figure 1(b)). No significant inhibition of hESC attachment to Matrigel was observed after blocking the *α*V subunit or the *β*5 subunit, in either 2% or 21% O_2_ (Figures 1(c) and 1(d)). However, blocking the *α*V*β*5 integrin demonstrated a significant reduction in hESC attachment to Matrigel in 2% O_2_ only (Figure 1(e)). Interestingly, blockage of CD44 receptor showed a significant reduction in hESC attachment to Matrigel in 21% only (Figure 1(f)). Data normalisation to control cultures (calculated by dividing concentration/dosage value by control value to establish comparative values) confirmed that statistically significant reductions in hESC attachment occurred in 2% O_2_ after blocking *α*V*β*5 and *α*6 (*P* < 0.05), and hESC attachment in 21% O_2_ was only inhibited after blocking CD44 (*P* < 0.05). Blocking of *α*E inhibited hESC attachment in both 2% and 21% O_2_ (*P* < 0.001) (Figure 1(g)). Due to the robust and distinct impacts on adherence of both *α*V*β*5 and CD44, we next sought to determine if transcriptional alterations were reflected at the translational level.

### 3.3. *α*V*β*5 and CD44 Receptor Immunofluorescence and Flow Cytometry Analysis

**Figure 2:**
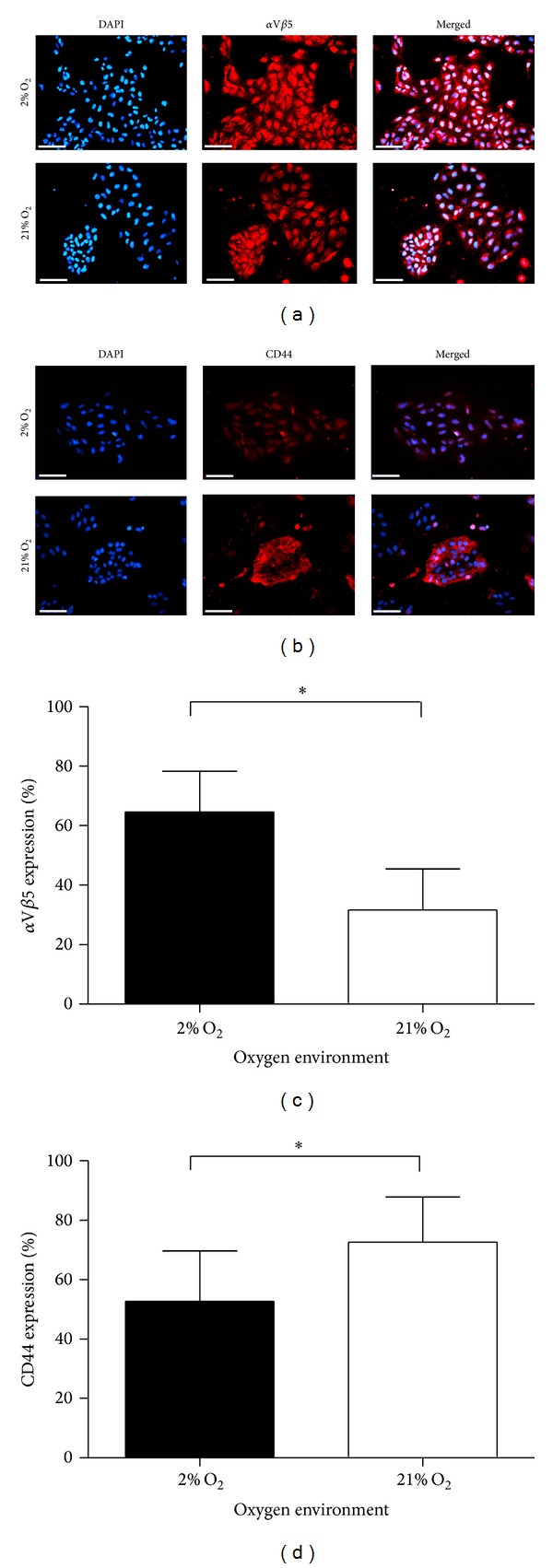
*α*V*β*5 and CD44 expressions in hESC. (a) Immunoflourescent staining of *α*V*β*5 integrin and (b) CD44 (HCAM) in hESCs cultured in both 2% O_2_ and 21% O_2_. Scale Bar = 100 *μ*m. Flow cytometry quantification of (c) *α*V*β*5 integrin expression and (d) CD44 receptor expression, in hESCs cultured in both 2% and 21% O_2_ environments. (*n* = 5); *P < 0.05. *y*-axis indicates % of hESCs with positive receptor expression. Black and open bars indicate 2% O_2_ and 21% O_2_, respectively. Error bars indicate standard deviations (SD).

Positive expression of *α*V*β*5 and CD44 receptors was immediately apparent in both O_2_ concentrations at apparently differing levels (Figures 2(a) and 2(b)). In 21% O_2_, the expression of CD44 appeared predominantly membrane bound and less abundant in the cytoplasmic and nuclear regions, whereas the converse was apparent in 2% O_2_ cultured cells (Figure 2(b)). The expression pattern of *α*V*β*5 in 2% O_2_ and 21% O_2_ appeared broadly similar with stronger staining in the former. Flow cytometry analysis revealed a 2-fold increase in *α*V*β*5 expression in hESCs cultured in 2% O_2_ (64.5%) compared to 21% O_2_ (32%) (*P* < 0.029) (Figure 2(c)). In support of our earlier observation, we also noted that a significantly higher percentage of hESCs cultured in 21% O_2_ (72.6%) expressed CD44 (1.4-fold) relative to 2% O_2_ cultured cells (52.7%) (*P* > 0.037) (Figure 2(d)).

### 3.4. Pluripotent Marker Expression in *α*V*β*5 and CD44 Blocked hESCs

**Figure 3:**
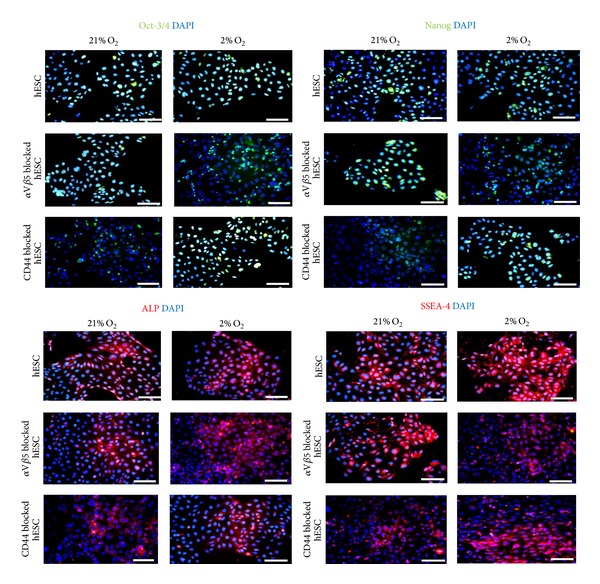
Pluripotent marker expression in *α*V*β*5 blocked hESCs and CD44 blocked hESCs. Immunoflourescent staining of pluripotent marker expression (Oct-3/4, Nanog, ALP, and SSEA-4) in hESCs, 24 hours after *α*V*β*5 blockage and CD44 blockage when cultured in 2% O_2_ or 21% O_2_. Nuclei are counterstained with DAPI (indicated in blue). Scale bar = 100 *μ*m.

**Figure 4:**
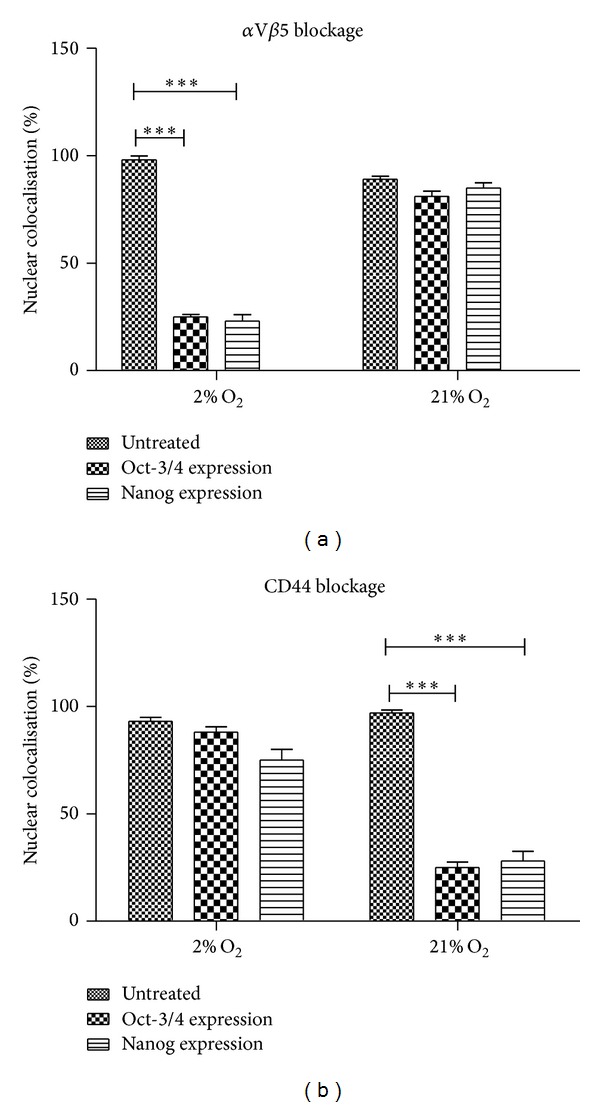
Nanog and Oct-4 nuclear colocalisation after surface receptor blockage. Quantification of nuclear-associated expression of Oct-4 and Nanog expression in hESCs which retained substrate adhesion after (a) *α*V*β*5 blockage in either 2% O_2_ or 21% O_2_ cultured hESC and (b) CD44 blockage in either 2% O_2_ or 21% O_2_ cultured hESC. ***P < 0.001. *y*-axis indicates % of nuclear colocalisation.

Immunostaining demonstrated that hESCs which retained a capacity for substrate adhesion after either *α*V*β*5 or CD44 blockage in hESCs cultured in 2% O_2_ or 21% O_2_ had distinct effects on their pluripotent marker location. Adhered hESCs after *α*V*β*5 blockage in 21% O_2_ retained nuclear colocalisation of Oct-4 and Nanog (Figure 3), whereas, in 2% O_2_, a 3.44-fold reduction in nuclear colocalisation of Oct-4 and a 3.63-fold reduction for Nanog were apparent (Figures 3 and 4). Similarly, an overall decrease in cytoplasmic expression of ALP and SSEA-4 was noted for hESC which had retained a capacity for substrate adherence in 2% O_2_ in comparison to 21% O_2_ after *α*V*β*5 blockage (Figure 3).

hESC which retained adherence after antibody blockage of CD44 in 2% O_2_ retained nuclear colocalisation of Oct-4 and Nanog, whereas, in 21% O_2_, a 3.62-fold decrease in nuclear colocalised Oct-4 and a 3.4-fold decrease in Nanog were observed (Figures 3 and 4). Furthermore, an apparent overall decrease in expression levels of ALP and SSEA-4 was also observed in adhered hESCs, cultured in 21% O_2_, in comparison to 2% O_2_ after CD44 blockage (Figure 3).

## 4. Discussion

Pluripotentiality, self-renewal, and relative ease of scale-up of hESC represent the main drivers behind regenerative medicine industry association and potential applicability in widespread disease treatments. Limitations behind immediate application remain and include tumorigenicity, xenogenic risk, and poorly defined mechanisms of action. Reduction of these limitations is associated with better understanding of the mechanisms of substrate adhesion. We have revealed that hESC substrate adhesion operates through integrin dependent and independent pathways where oxygen tension plays a key role in mechanism choice with implications for pluripotential retention.

Integrin receptors expressed by hESC play a vital role in adhesion to ECM proteins such as laminin (*α*6*β*1), vitronectin (*α*V*β*5) and fibronectin (*α*V*β*1, *α*5*β*1), and collagen and laminin (*α*2*β*1), nidogen, laminin, collagen 1 and fibronectin (*α*3*β*1), collagen (*α*11*β*1) [8, 17, 18]. It is evident, therefore, that substantial redundancies exist across the signalling and cell-matrix interaction pathways that are associated with hESC adhesion and self-renewal. Previous reports have detailed transcriptional alterations resulting in reduced transcriptional heterogeneity following culture of hESC in a reduced oxygen environment [30, 34]; this includes a study by our group which specifically revealed that integrin subunits, *α*5 (*P* < 0.01) and *α*6, *α*E, *α*V, and *β*5 (all *P* < 0.001), were expressed significantly higher in hESCs cultured in 2% O_2_ when compared to 21% O_2_ (see Supplementary Figure  1 and Supplementary Table  1) [31]. Furthermore, previous reports have detailed a reliance on *α*V*β*5 and *α*6 integrin subunits for hESC attachment in 21% O_2_ [7, 8], which differs from our observations where significant alterations in integrin gene expression, attachment rates, and surface-bound receptor were observed in 2% O_2_ only. However, the remit of these previous investigations was not to compare the effects of different oxygen environments on the reliance of *α*V*β*5 and *α*6 integrin subunits for hESC attachment and thus may have observed even greater significant differences when investigated under 2% O_2_. The concentration of antibody blocking solution used for *α*6 in the study by Meng et al., 2010, was also much higher (10 mg/mL) in comparison to the concentration range used in this study (0–50 *μ*g/mL) [7]. In our study, we did notice variation in control attachment numbers for untreated cells (Figures 1(a)–1(f)). In our experience, multiple factors can affect attachment including Matrigel and conditioned media used at the time of each experiment. For example, variations in Matrigel lot or MEF-conditioned media batch could result in different concentrations of proteins relative to each other, which could subsequently have an effect on the availability of appropriate ligands for corresponding hESC surface integrin receptors. For this reason, all attachment data was performed with an *n of 3* with each *n* incorporating 3 experimental repeats normalised to the corresponding control attachment values for each integrin data set, in order to validate the significance in the change of integrin expression as a result of oxygen environment.

CD44 is a specific receptor and mediator for hyaluronic acid (HA), which promotes hESC proliferation and associated intracellular pathways [11, 32]. HA, secreted by MEFs (feeder cells) into media at a concentration of approximately 840 ng/mL, plays a critical role in coregulation of gene expression, signalling, proliferation, motility, and adhesion of hESCs where levels are higher in undifferentiated hESCs and decrease with onset of differentiation [11, 35]. Our results provide validation and extension of recent reports in which antibody blocking of CD44 was described as reducing hESC clonogenicity in 21% O_2_ [11, 35]. Our previous study also noted the significant upregulation of HA-associated genes; Hyaluronan and proteoglycan link protein 3, Hyaluronan-mediated motility receptor, and Hyaluronoglucosaminidase 2 in 21% O_2_ (see Supplementary Table  1). Taken together with our previous observations, these data strongly suggest that oxygen signalling has a role in defining substrate adhesion mechanistic choice. In hypoxia, there is a downregulation in the expression of hyaluronic acid associated genes: and blockage of the CD44 receptor in 2% O_2_ had little effect on cell attachment. This demonstrates the clear switch in the reliance of a specific receptor for hESC attachment in different oxygen environments, in this case, being CD44 in 21% O_2_ to *α*V*β*5 in 2% O_2_. Although microarray data has demonstrated that transcription of CD44 is not hypoxia repressible, it appears that translation of CD44 is repressible suggesting that the message is not being relayed into a protein, in 2% O_2_. Further studies are required to elucidate the precise mechanisms underlying substrate choice pathways.

Interference with substrate adhesion mechanisms had an immediate role in maintenance of the undifferentiated state in hESCs. Oxygen itself is a bioactive, signalling molecule which, in conjunction with other regulatory factors, can influence various cellular activities including cell attachment and proliferation as well as intracellular pathways which are involved in controlling stemness. Hypoxia inducible factors interact with integrins and growth factor signalling which are also strongly interlinked and act in combination. The activation of these pathways is crucial in maintaining pluripotency of hESCs, through mechanisms which are yet to be clearly defined [35]. Antibody blockage of *α*V*β*5 (in 2% O_2_) and CD44 (in 21% O_2_) significantly decreased the nuclear localisation of Oct-3/4 and Nanog which strongly suggests an oxygen-linked substrate adhesion mechanism choice pathway. To function as pluripotency regulators, Nanog and Oct-3/4 require nuclear colocalisation [36]. In this instance, nuclear localisation was lost as a direct consequence of substrate adhesion receptor blockage in an oxygen-specific manner. In addition to these, a substantial decrease in Alkaline phosphatase and SSEA-4 expression was also noted in both. The consistency of response indicates that either *α*V*β*5 or CD44 is signalling via similar pathways, for instance, interfering with FGF-2 signalling pathway resulting in inactivation of pathways MAPK/ERK, PI3/AKT kinase, and NF_k_B or through distinct, though mechanistically identical, self-renewal maintenance pathways [37, 38]. More specifically, in 2% O_2_, HIFs are able to activate signalling pathways including FGF and notch through upregulating the expression of transcriptional factors such as NF_k_B, activator protein-1, p53, and C-myc [39, 40]. Therefore, it is apparent that the inhibition of receptor *α*V*β*5 results in the outside-in signalling effect resulting in the inactivation of these intracellular pathways which cause the inactivation in the expression of pluripotent genes. As for CD44, we believe that blocking this receptor in 21% O_2_ interferes with the FGF-2 signalling pathway which is known to cause inactivation of MAPK, ERK, PI3/AKT kinase, and NFKB pathways which has been evidently shown to cause a downregulation of pluripotent markers.

Recognition of the specific integrin mediators and ECM proteins, required for hESC attachment and growth whilst retaining pluripotency, is fundamental for scaling up culturing protocols for potential therapeutic applications. Concurrent research has attempted to implement these findings into designing or investigating alternate defined substrates to Matrigel for hESC culture coupled to elimination of xenogenic contamination. Recent studies have defined individual ECM proteins such as laminin, vitronectin, fibronectin, and collagen as alternative hESC culture substrates [8]. Uniquely, we have demonstrated in this study that oxygen is an additional factor that drives the substrate adhesion ability of hESCs. The definition of essential hESC integrin receptors in relation to oxygen will enable the effective and constructive design of novel engineered substrates (synthetic or natural) which would present tailored ligand sites for corresponding hESC receptors resulting in enhanced hESC attachment and self-renewal at an enhanced rate with the elimination of xenogenic substrates. The development of defined substrates will enhance the definition and standardisation of hESC culture and assist in clinical translation of hESC products.

## 5. Conclusion

hESC substrate-attachment mechanisms are related to the oxygen environment in which hESCs are cultured. This study describes the specific, key integrins and adhesion molecules which mediate this function in an oxygen concentration-dependent manner. These data will assist in the design of novel substrates with the potential to eliminate xenogenic substrate components and promote hESC adhesion in the relevant oxygen environments. The optimised oxygen environment, nonbiologicial xeno-free substrate, and defined media would result in a simpler, *in vitro* hESC culture technique and improve the scale-up of hESCs, which could provide exciting opportunities for *in vivo* cell therapy and regenerative medicine applications.

## Supplementary Material

Microarray data analysis revealed relative expression patterns of selected integrin sub-units in hESCs cultured in 2% and 21% O_2_ conditions.
