# Supplementary material for: αVβ5 and CD44 Are Oxygen-Regulated Human Embryonic Stem Cell Attachment Factors
Source: Biomed Res Int. 2013 Dec 26;2013:729281. doi: 10.1155/2013/729281 (PMC3888678; doi:10.1155/2013/729281)
Supplement: Supplementary file 1 — Microarray data analysis revealed relative expression patterns of selected integrin sub-units in hESCs cultured in 2% and 21% O2 conditions. [file 729281.f1.pdf]

Supplementary file: Deepak Kumar Integrin Manuscript

In silico microarray analysis of a previously published dataset utilising hESC (H1, H9, and RH1) cultured in both 21% O_2 and 2% O_2 was conducted as previously described (44). We note that these are different hESC lines to the one used for all experiments conducted in this study however we, and others, have demonstrated that culture in reduced oxygen resulted in reduced transcriptional heterogeneity between hESC lines thus validating our use of an independent hESC line (45). Data was then sorted by gene name, relative expression values of <10 removed, and multiple probe hits deleted after generating averaged values.

Supplementary Table 1. Microarray signal intensity values for all integrins and CD44-related genes in hESCs cultured in physiological normoxia (2% O2) and hyperoxia (21% O2). Normalisation was performed against baseline signal using the D Chip platform as described in Forsyth et al 2008 (44).

**Gene Symbol (Gene Name)**	**2% O_2**	**21% O_2**	**P Value**	**Fold Change**
ITGB1BP2 (Integrin β1 binding protein 2)	16.25	11.79	0.14	1.38
AaeL-AAEL007077 (Integrin β1 binding protein 1)	212.15	177.21	$7x10^{-3}$	1.20
ITGB1BP3 (Integrin β1 binding protein 3)	496.54	544.12	0.47	1.10
ITGB3BP (Integrin β3 binding protein)	595.30	545.43	$3\ x10^{-3}$	1.09
LOC658655 (Integrin β4 binding protein)	1354.68	1074.23	0.02	1.26
ITGA1 (Integrin α1)	25.87	27.10	0.77	1.05
ITGA10 (Integrin α10)	39.93	34.89	0.43	1.14
ITGA11 (Integrin α11)	88.46	91.98	0.65	1.04
ITGA2 (Integrin α2)	21.55	28.47	0.24	1.32

ITGA2B (Integrin α2b)	64.27	67.22	0.63	1.05
ITGA4 (Integrin α4)	23.37	18.80	0.35	1.24
ITGA5 (Integrin, α5)	182.51	134.33	0.04	1.36
ITGA6 (Integrin α6)	1229.82	938.91	2×10^{-4}	1.31
ITGA7 (Integrin α7)	163.05	117.39	0.24	1.39
ITGA8 (Integrin α8)	34.53	43.15	0.09	1.25
ITGA9 (Integrin α9)	43.93	28.54	0.01	1.54
ITGAD (Integrin αD)	20.82	11.50	0.05	1.81
ITGAE (Integrin αE)	553.67	423.84	3×10^{-4}	1.31
ITGA11 (Integrin L)	16.06	15.69	0.88	1.02
ITGAM (Integrin αM)	21.03	19.04	0.48	1.11
ITGAV (Integrin αV)	851.72	524.89	1×10^{-4}	1.64
ITGB1 (Integrin β1)	4691.45	3910.88	0.03	1.20
ITGB2 (Integrin β2)	47.30	51.05	0.53	1.08
ITGB3 (Integrin β3)	45.74	36.88	0.25	1.24
ITGB4 (Integrin β4)	51.26	46.43	0.26	1.10
ITGB5 (Integrin β5)	1927.06	1662.95	1×10^{-3}	1.16
ITGB6 (Integrin β6)	6.52	12.10	0.11	1.86

ITGB7 (Integrin β7)	25.14	21.48	0.42	1.17
ITGB8 (Integrin β8)	17.36	16.89	0.82	1.03
ITGBL1 (Integrin β-like 1)	36.32	34.09	0.39	1.07
IIK (Integrin-linked kinase)	796.20	756.35	0.38	1.05
IIKAP (Integrin-linked kinase-associated serine/threonine phosphatise 2C)	191.87	183.45	0.31	1.05
CD44 molecule (Indian blood group)	54.08	42.64	0.37	1.27
HAPLN3 (Hyaluronan and proteoglycan link protein 3)	87.09	78.79	0.05	0.90
HMMR (Hyaluronan-mediated motility receptor, RHAMM)	269.57	385.62	$3x10^{-5}$	1.43
HYAL2 (Hyaluronoglucosaminidase 2)	415.54	519.13	$9x10^{-3}$	1.25

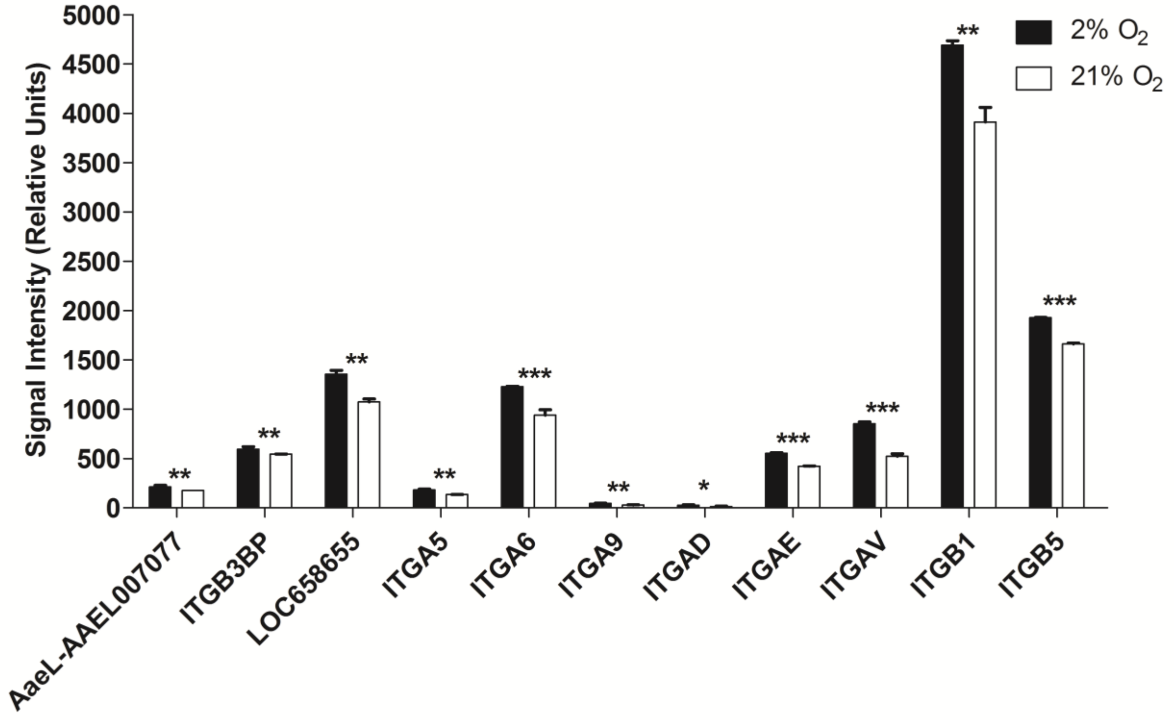

Supplementary Figure 1. Integrin blocking effects on hESCs attachment

hESCs cultured in 2% O_2 or 21% O_2 and pre-incubated with anti- (A) α6, (B) αE, (C) αV, (D) β5, (E) αVβ5 and (F) CD44 antibodies. (n=6); * $p < 0.05$, ** $p < 0.01$, *** $p < 0.001$. y-axis indicates % of input cell attachment post-antibody blocking after 24 hours. Black and open bars indicate % of cell attachment in 2% O_2 and 21% O_2 after antibody blocking treatment, respectively. Error bars indicate standard deviations (SD). (G) Cell attachment data normalised to unblocked controls. Asterisks indicate significant differences to unblocked controls. Values indicate mean percentage of cell attachment (n=6); * $p < 0.05$, ** $p < 0.01$ and *** $p < 0.001$, a <0.05 and b<0.01 indicate significant differences due to oxygen tension (comparison between 21% O_2 and 2% O_2 for each integrin sub-unit). Error bars indicate standard error (SE).
